# Supplementary material for: A Multi-Trait Approach Identified Genetic Variants Including a Rare Mutation in RGS3 with Impact on Abnormalities of Cardiac Structure/Function
Source: Sci Rep. 2019 Apr 10;9:5845. doi: 10.1038/s41598-019-41362-3 (PMC6458140; doi:10.1038/s41598-019-41362-3)
Supplement: Supplementary file 1 — Supplementary [file 41598_2019_41362_MOESM1_ESM.pdf]

A Multi-Trait Approach Identified Genetic Variants Including a Rare Mutation in RGS3 with Impact on Abnormalities of Cardiac Structure/Function

Akram Yazdani*, Azam Yazdani, Raúl Méndez Giráldez, David Aguilar, Luca Sartore

Supplementary Figures

Supplementary Figure 1

The histogram of the traits in Table1 after winsorization, standardization, and normal transformation

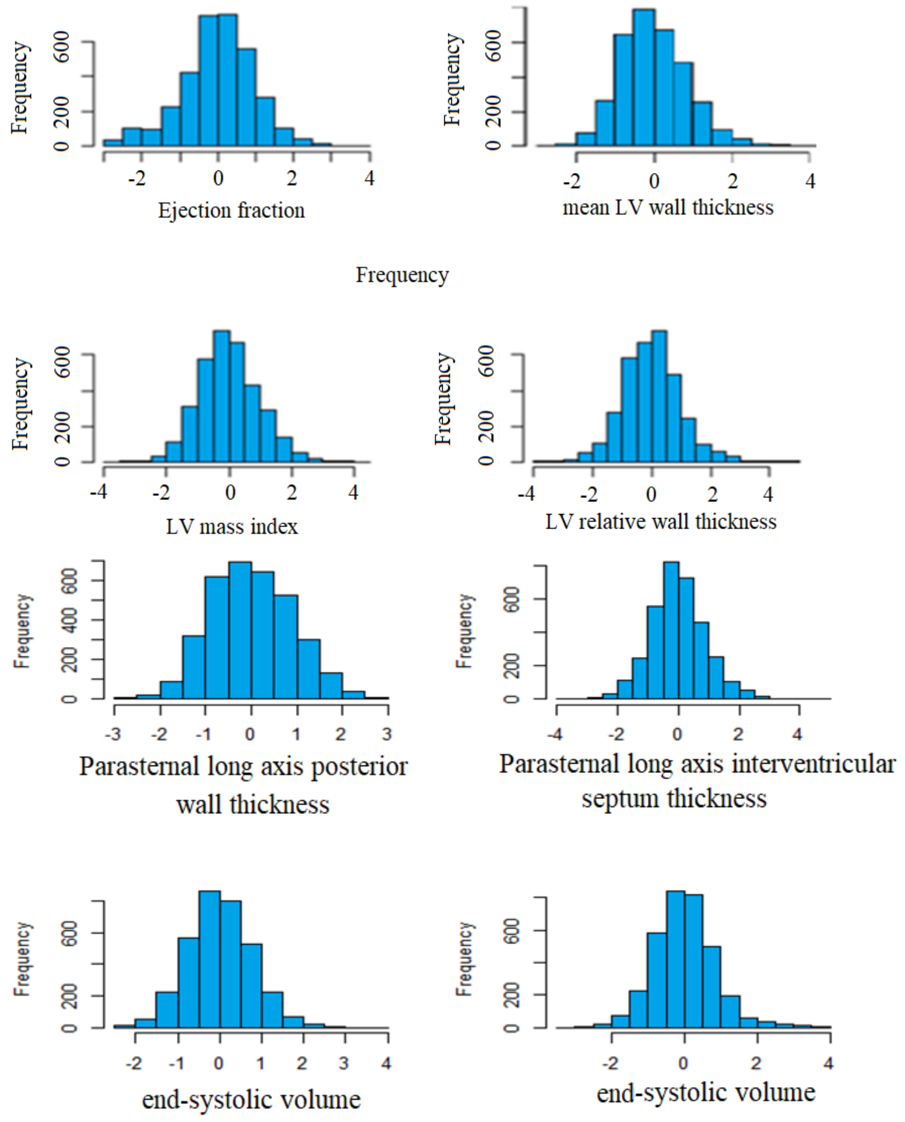

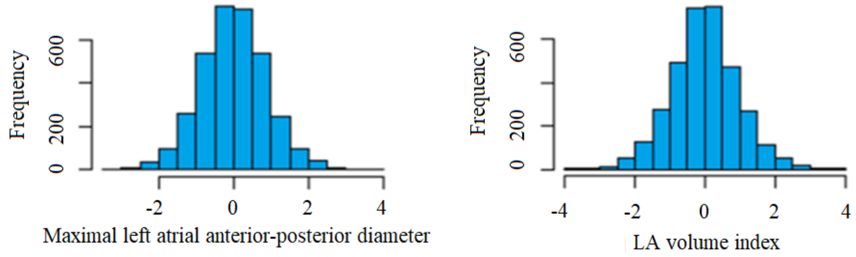

Supplementary Figure 2

Empirical Distributions of the traits in Table 3 for individuals with Reference allele: The yellow vertical line shows the third quartiles of the distribution. Individuals with alternative allele are represented with blue vertical line.

NC_000009.11:g.116346115C>A in *RGS3*

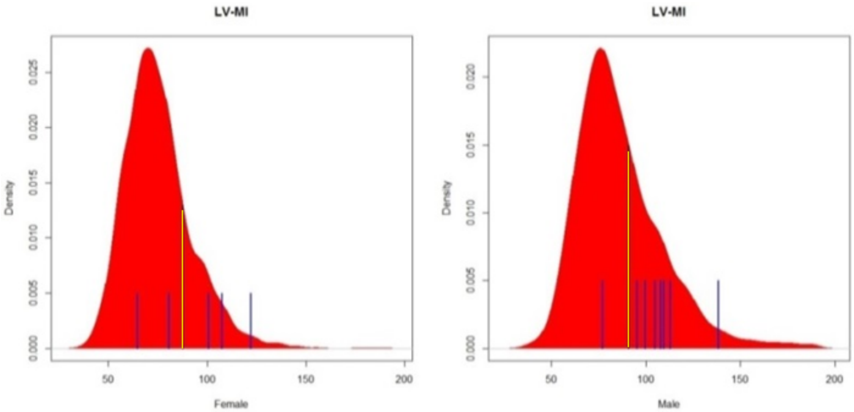

NC_000009.11:g.116346115C>A in *RGS3*

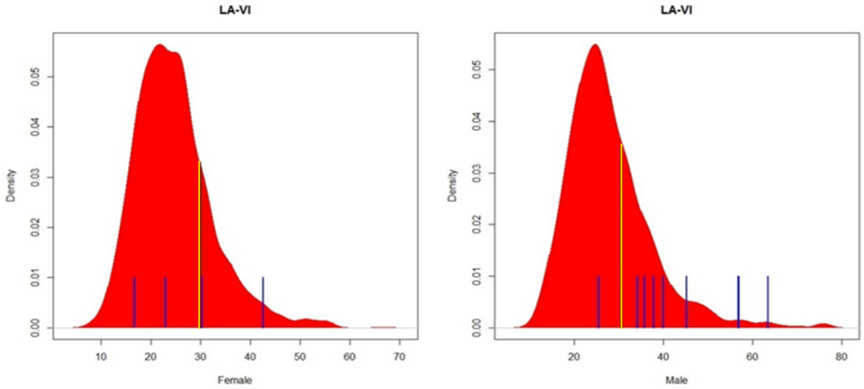

NC_000009.11:g.116346115C>A in *RGS3*

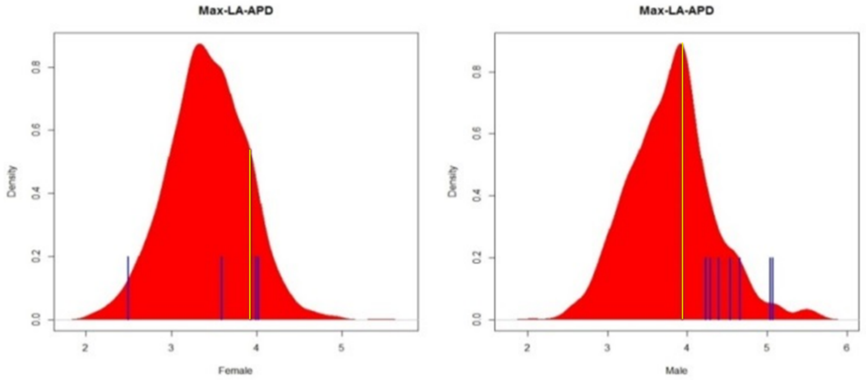

NC_000017.10:g.7802658C>T in CHD3

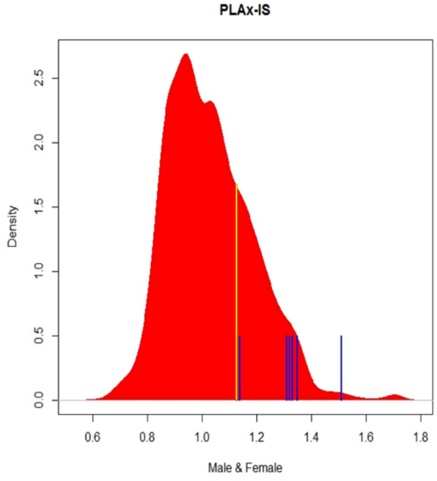

NC_000017.10:g.73897977C>T in *MRPL38*

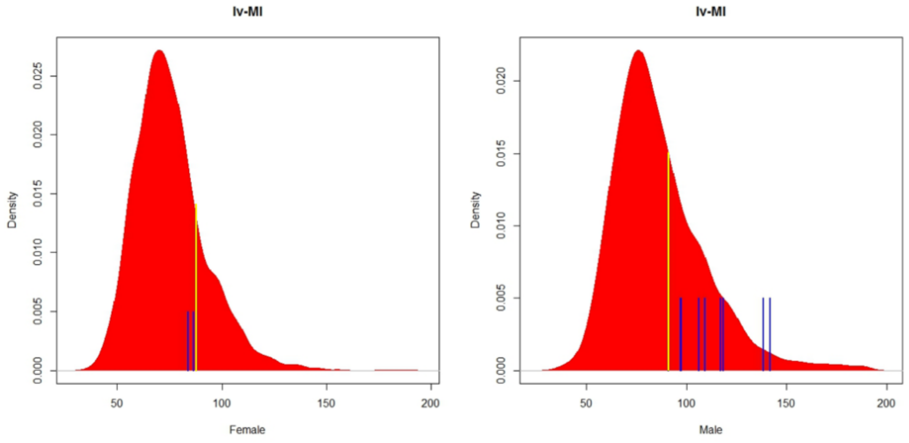

Supplementary Tables

The VEP analysis results for identified genetic variants are summarized in the following tables where

- **HGVS name**: description of sequence variation in genomic established by the Human Genome Variation Society
- **Consequence:** consequence type of the allele on transcript
- **HGVSp:** HGVS protein sequence name
-

Supplementary Table 1

HGVS name	Allele	Consequence	Gene symbol	Exon	Intron	HGVSp	Amino acids	Codons
NC_000009.11: g.116346115C>A	A	missense_variant	RGS3	2/7	-	NP_001263189.1: p.Thr129Asn	T/N	aCc/aAc
NC_000009.11: g.116346115C>A	A	intron_variant	RGS3	-	10/14	-	-	-
NC_000009.11: g.116346115C>A	A	missense_variant	RGS3	3/8	-	NP_001269851.1: p.Thr129Asn	T/N	aCc/aAc
NC_000009.11:g. 116346115C>A	A	missense_variant	RGS3	18/23	-	NP_001269852.1: p.Thr698Asn	T/N	aCc/aAc
NC_000009.11: g.116346115C>A	A	missense_variant	RGS3	2/7	-	NP_001309144.1: p.Thr147Asn	T/N	aCc/aAc
NC_000009.11: g.116346115C>A	A	missense_variant	RGS3	11/16	-	NP_570613.2: p.Thr527Asn	T/N	aCc/aAc
NC_000009.11: g.116346115C>A	A	intron_variant	RGS3	-	1/4	-	-	-
NC_000009.11: g.116346115C>A	A	missense_variant	RGS3	21/26	-	NP_652759.3: p.Thr808Asn	T/N	aCc/aAc
NC_000009.11: g.116346115C>A	A	non_coding_ transcript_ exon_variant	RGS3	18/23	-	-	-	-
NC_000009.11: g.116346115C>A	A	non_coding_ transcript_ exon_variant	RGS3	3/8	-	-	-	-

Supplementary Table 2

HGVS name	Allele	Consequence	Gene symbol	Biotype	Exon	Intron	HGVSp	Amino acids	Codons
NC_000017.10: g.7802658C>T	T	splice_region_ variant,intron_ variant	CHD3	protein_ coding	-	14/39	-	-	-
NC_000017.10: g.7802658C>T	T	splice_region_ variant,intron_ variant	CHD3	protein_ coding	-	14/39	-	-	-
NC_000017.10: g.7802658C>T	T	splice_region_ variant,intron_ variant	CHD3	protein_ coding	-	14/38	-	-	-
NC_000017.10: g.7802658C>T	T	splice_region_ variant,intron_ variant	CHD3	protein_ coding	-	14/39	-	-	-
NC_000017.10: g.7802658C>T	T	splice_region_ variant,intron_ variant	CHD3	protein_ coding	-	14/38	-	-	-
NC_000017.10: g.7802658C>T	T	splice_region_ variant,intron_ variant	CHD3	protein_ coding	-	14/39	-	-	-
NC_000017.10: g.7802658C>T	T	splice_region_ variant,intron_ variant	CHD3	protein_ coding	-	14/33	-	-	-
NC_000017.10: g.7802658C>T	T	splice_region_ variant,intron_ variant	CHD3	protein_ coding	-	14/21	-	-	-

Supplementary Table 3

HGVS name	Allele	Consequence	Gene symbol	Biotype	Exon	Intron	HGVSp	Amino acids	Codons
NC_000017.10: g.73897977C>T	T	upstream_gene_ variant	TRIM65	protein_ coding	-	-	-	-	-
NC_000017.10: g.73897977C>T	T	missense_variant	MRPL38	protein_ coding	4/9	-	-	R/Q	cGg/cAg
NC_000017.10: g.73897977C>T	T	upstream_gene_ variant	TRIM65	protein_ coding	-	-	-	-	-
NC_000017.10: g.73897977C>T	T	non_coding_ transcript_ exon_variant	MRPL38	misc_RNA	4/8	-	-	-	-

Supplementary statistical methods

Let assume that we measured q phenotypic traits for n individuals with p recorded genotypic variants. The multi-trait polygenic mixed model for this data can be represented as

$$\boldsymbol{Y}_{\mathrm{nq}\times 1} = \boldsymbol{\mu}_{\mathrm{nq}\times 1} + \left(\boldsymbol{X}_{\mathrm{n}\times\mathrm{p}} \otimes \boldsymbol{I}_{\mathrm{q}}\right)\boldsymbol{\beta}_{\mathrm{pq}\times 1} + \boldsymbol{U}_{\mathrm{nq}\times 1} + \boldsymbol{\epsilon}_{\mathrm{qn}\times 1} \qquad (1)$$

where

$$\boldsymbol{\epsilon} \sim N(\boldsymbol{0}, \Sigma),\ \Sigma_{nq\times nq} = \mathrm{diag}[\Sigma_{ii}]_{q\times q},\ \Sigma_{ii} = \mathrm{diag}[\sigma_i]_{n\times n}.$$

Each entry of $\boldsymbol{Y} = \{y_{ij}\}_{\substack{i=1,\dots,q \\ j=1,\dots,n}}$ represents ith trait recorded for jth individual, β_{ik} is the effect of kth genomic variants on ith trait in coefficient vector $\boldsymbol{\beta} = \{\beta_{ik}\}_{\substack{i=1,\dots,q \\ k=1,\dots,p}}$, vector $\boldsymbol{U} = \{u_{ij}\}_{\substack{i=1,\dots,q \\ j=1,\dots,n}}$ includes random effects corresponding to vector Y as

$$\boldsymbol{U} \sim N(\boldsymbol{0}, \Psi)$$

where

$$\Psi = \begin{bmatrix} \Psi_{11} & \cdots & \Psi_{1q} \\ \vdots & \ddots & \vdots \\ \Psi_{q1} & \cdots & \Psi_{qq} \end{bmatrix},$$

and $\Psi_{il} = \{\psi_{jg}\}_{\substack{j=1,\dots n,\\ g=1,\dots,n}}, \quad i, l \in \{1, \dots, q\}$.

Without loss of generality, we assume y_{ij}s are standardized and write the likelihood function as

$$\ell(\boldsymbol{\beta}, \boldsymbol{U}, \Sigma, \Psi \,|\boldsymbol{Y}) \propto \exp\left\{-\frac{1}{2}\, \boldsymbol{e}^T \Sigma^{-1} \boldsymbol{e}\right\}$$

where $\boldsymbol{e} = \boldsymbol{Y} - (\boldsymbol{X} \otimes \boldsymbol{I}_q)\boldsymbol{\beta} - \boldsymbol{U}$.

Bayesian network over traits: The large sample size (*n*) and large number of traits (*q*) introduce a large number of parameters into the model through Ψ and make the numerical algorithm of model (1) unstable and slow to converge. Therefore, to reduce the number of parameters in multi-trait polygenic mixed model and provide larger precision for parameter estimations, we propose to estimate the sparse structure of precision matrix Ψ^{-1} in priori using phenotypic data (individuals without genotype record) which is accessible in majority of medical studies. Here in particular, we apply a Bayesian network that relies on probabilistic graphical models to estimate sparse relationship among traits.

Bayesian networks represent underlying relationship among traits based on efficient and effective representation of their joint probability distribution (1)(2)(3) where nodes represent traits and links represent partial correlations. A missing edge between two traits reveals that the two corresponding traits do not have a significant relationship after excluding effect of the other traits in the analysis. To learn about sparsity of precision matrix Ψ^{-1}, we incorporate independencies inferred from Bayesian networks to reduce the number of parameters in the model and efficiently compute posterior probabilities. In the matrix Ψ^{-1}, we replace zero with the parameters corresponding to missing arrows in the identified Bayesian network over the traits. To avoid overfitting, we identify the Bayesian network using the data on the set of individuals without genotype record.

Prior Specification: There have been many studies on setting a prior distribution on precision matrix Ψ^{-1}. The most common approach is to set Wishart distribution as prior on Ψ^{-1} while it is conjugate prior for multivariate normal model. Wishart distribution is fully parameterized with a single degree of freedom parameter and scale matrix parameter. The degree of freedom parameter that needs to be larger than dimension of relatedness matrix (Ψ) represents the strength of information surrounded around scale matrix parameter. Therefore, in large scale problems, this choice of prior yields to highly concentrated distribution about the scale matrix(4)(5)(6).

We here assume that individuals are independent which can be readily satisfied in population studies and clinical studies that are not family based. The assumption of independent individuals leads to a sparse relatedness matrix Ψ as

$$\Psi = \begin{bmatrix} \Psi_{11} & \cdots & \Psi_{1q} \\ \vdots & \ddots & \vdots \\ \Psi_{q1} & \cdots & \Psi_{qq} \end{bmatrix}, \qquad \Psi_{il} = \mathrm{diag}\left[\psi_{jg}\right], \ \ i, l \in \{1, \ldots, n\}, \ \ j, g \in \{1, \ldots, q\}.$$

To set prior on this matrix, we first rearrange vector **U** in order to be partition with individual's index *j*. If we denote the rearranged random effect vector with $\boldsymbol{U}^*$ such that $\boldsymbol{U}^* \sim \mathrm{N}(\mathbf{0}, \Psi^*), \Psi^*$ is a block diagonal as

$$\Psi^* = \begin{bmatrix} \Psi_{11}^* & \cdots & 0 \\ \vdots & \ddots & \vdots \\ 0 & \cdots & \Psi_{nn}^* \end{bmatrix}$$

where each Ψ_{jj}^* is a $q \times q$ dense matrix. Ψ_{jj}^* takes into account correlation among different traits for *j*th individual. While Ψ^{*-1} is a block diagonal matrix and q is a small number, the use of Wishart prior for $\Psi_{jj}^{*-1}$is appropriate. Furthermore, we incorporate the identified relationship among the traits using Bayesian network and estimate the sparsity of $\Psi_{jj}^{*-1}$and set G-Wishart (GW) distribution (7) as prior on Ψ_{jj}^{*-1}. G-Whishart distribution, which is a restricted domain of

Wishart given constraints of network structure, provides sparse block diagonal matrix and reduces computational burden time. The density of G-Wishart is

$$p\left(\Psi_{jj}^{*-1} | \mathrm{BN_T}\right) = \left(I_{G_T}(\nu, \Lambda)\right)^{-1} \left|\Psi^{*-1}{}_{jj}\right|^{\frac{\nu-2}{2}} \exp\left\{-\frac{1}{2}\mathrm{tr}\left(\Lambda\Psi^{*-1}{}_{jj}\right)\right\}$$

where $\mathrm{BN_T}$ stands for the Bayesian Networks over the traits, and

$$I_{GT}(\nu, \Lambda) = \int |\Psi_{jj}^{*-1}|^{(\nu-2)/2} \exp\left\{-\frac{1}{2}\mathrm{tr}\left(\Lambda\Psi_{jj}^{*-1}\right)\right\} \mathrm{d}\Psi_{jj}^{*-1}$$

is the normalizing constant, which is finite for $\nu > 2$ .This results in a reduction of the number of parameters in multi-trait polygenic mixed model, and consequently expedites the convergence of the algorithm.

We set conjugate prior for other hyperparameters of the model as

$$\boldsymbol{\beta} \sim \mathrm{N}\ (0, \Omega)$$

where

$$\Omega_{pq\times pq} = \mathrm{diag}\ [\Omega_{kk}]_{p\times p}, \qquad \Omega_{kk} = \mathrm{diag}\ \left[\sigma_j\right]_{p\times p}$$

and

$$\sigma_i \sim IG\ \left(\frac{a_1}{2}, \frac{b_1}{2}\right)$$

The aforementioned prior specification leads to Gibbs sampling scheme for the model with the graphical representation shown in the following Figure.

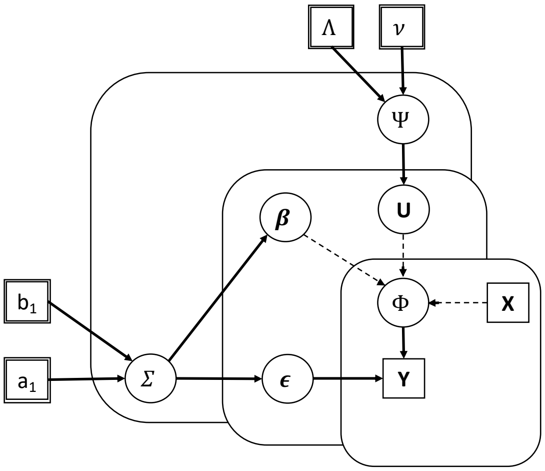

Figure: Graphical representation of the model; $\boldsymbol{Y} = \Phi + \boldsymbol{\epsilon}$ and $\Phi = (\boldsymbol{X} \otimes I)\boldsymbol{\beta} + \boldsymbol{U}$

In the graphical representation of the model, circle nodes represent unobserved stochastic variables, square nodes with a single border represent observed stochastic variables (i.e., data), squares with double border denote fixed quantities in prior distributions. The arrows represent the dependencies between variables.

Gibbs Sampling Scheme of IBMT:

$$\boldsymbol{\beta}_i \mid \boldsymbol{y}_i, \boldsymbol{u}_i, \boldsymbol{\sigma}_i \sim \mathrm{N}\,[\,(\boldsymbol{X}^T\boldsymbol{X} + \boldsymbol{I})^{-1}\boldsymbol{X}^{-1}(\boldsymbol{y}_i - \boldsymbol{u}_i), \sigma_i(\boldsymbol{X}^T\boldsymbol{X} + \boldsymbol{I})^{-1}]$$

$$\mathbf{u}_j^* \mid \mathbf{z}_j, \Psi_{jj}^*, \Sigma_{jj} \sim \mathrm{N}\left[\left(\Psi_{jj}^* + \Sigma_{jj}^{-1}\right)^{-1}\Sigma_{jj}^{-1}\mathbf{z}_j, \quad \left(\Psi_{jj}^* + \Sigma_{jj}^{-1}\right)^{-1}\right]$$

$$\sigma_i \mid \boldsymbol{y}_i, \boldsymbol{\beta}_i, \boldsymbol{u}_i \sim \mathrm{ING}\left[\frac{n+p+a_1}{2}, \frac{1}{2}(\boldsymbol{e}_i{}^T\boldsymbol{e}_i + \boldsymbol{\beta}_i^T\boldsymbol{\beta}_i + b_1)\right]$$

$$\Psi_{jj}^{*-1} \mid \boldsymbol{u}_j \sim G\mathrm{W}\left[\Lambda + \boldsymbol{u}_j^T\boldsymbol{u}_j, \quad \nu + q\right]$$

where $\boldsymbol{e_i} = (\boldsymbol{Y_i} - \boldsymbol{X\beta_i} - \boldsymbol{U_i})$ and vector Z is obtained after reordering entries of vector $(\boldsymbol{Y} - \boldsymbol{X} \otimes \boldsymbol{I}_q)\,\boldsymbol{\beta}$ in order to be partitioned by each individual similar to $\boldsymbol{U}^*$.

Supplementary references

1. Pearl J. Probabalistic Reasoning in Intelligent Systems. Probabalistic Reason Intell Syst. 1988;552.
2. Yazdani A, Yazdani A, Samiei A, Boerwinkle E. Erratum to: A causal network analysis in an observational study identifies metabolomics pathways influencing plasma triglyceride levels. J Biomed Inform. 2016;63:337–43.
3. Yazdani A, Yazdani A, Boerwinkle E. A Causal Network Analysis of the Fatty Acid Metabolome in African-Americans Reveals a Critical Role for Palmitoleate and Margarate. Omi A J Integr Biol. 2016;20(8):480–4.
4. Xiang R, Khare K, Ghosh M. High dimensional posterior convergence rates for decomposable graphical models. Electron J Stat. 2015;9(2):2828–54.
5. Banerjee S. Posterior convergence rates for high-dimensional precision matrix estimation using G-Wishart priors. Stat. 2017;6(1):207–17.
6. Gelman A. Prior distributions for variance parameters in hierarchical models (comment on article by Browne and Draper). Bayesian Anal. 2006;1(3):515–34.
7. Roverato A. Hyper Inverse Wishart Distribution for Non-decomposable Graphs and its Application to Bayesian Inference for Gaussian Graphical Models. Scand J Stat. 2002;29(1993):391–411.
